# Bereavement and Risk of Cardiovascular Disease Before and During the COVID-19 Pandemic

**DOI:** 10.1001/jamanetworkopen.2026.9102

**Published:** 2026-04-24

**Authors:** Fen Yang, Shiyu Li, Mary M. Barker, Huiqi Li, Krisztina D. László, Mikael Rostila, Sandra Rogne, Maria Feychting, Unnur A. Valdimarsdóttir, Fredrik Nyberg, Fang Fang

**Affiliations:** 1 Institute of Environmental Medicine, Karolinska Institutet, Stockholm, Sweden; 2 School of Public Health and Community Medicine, Institute of Medicine, Sahlgrenska Academy, University of Gothenburg, Gothenburg, Sweden; 3 Department of Global Public Health, Karolinska Institutet, Stockholm, Sweden; 4 Department of Public Health and Caring Sciences, Uppsala University, Uppsala, Sweden; 5 Department of Public Health Sciences, Stockholm University, Stockholm, Sweden; 6 Centre for Health Equity Studies, Stockholm University and Karolinska Institutet, Stockholm, Sweden; 7 Centre of Public Health Sciences, Faculty of Medicine, University of Iceland, Reykjavik, Iceland; 8 Department of Epidemiology, T.H. Chan School of Public Health, Harvard University, Boston, Massachusetts

## Abstract

**Importance:**

Bereavement is a known risk factor for cardiovascular disease (CVD), but it remains unclear whether the COVID-19 pandemic—which disrupted health care and increased social isolation—altered this association.

**Objective:**

To compare the association between bereavement and incident CVD before and during the pandemic.

**Design, Setting, and Participants:**

This cohort study of Swedish national health registries included individuals aged 30 years or older in Sweden during the pre–COVID-19 (2018-2019) and COVID-19 (2020-2021) periods. Analyses were performed between September 2024 and August 2025.

**Exposures:**

Bereavement exposure was defined as the loss of a partner, child, parent, or sibling.

**Main Outcomes and Measures:**

The first diagnosis of any CVD event, identified from an outpatient hospital visit or hospitalization via the Swedish Patient Register or death via the Swedish Cause of Death Register. Cox regression was used to estimate hazard ratios (HRs) of incident CVD after bereavement by study period, type of loss, and age.

**Results:**

Analysis included a total of 5 365 829 study participants (51.4% female; median [IQR] age, 51.6 [40.4-64.6] years) during the pre–COVID-19 period and 5 522 898 study participants (51.4% female; median [IQR] age, 49.8 [38.2-62.8] years) during the COVID-19 period. Overall, 372 477 (6.94%) and 368 902 (6.68%) incident CVD cases were identified during the pre–COVID-19 and COVID-19 periods, respectively. Bereavement was associated with increased CVD risk regardless of period or type of loss. However, the risk increment was greater during the COVID-19 period compared with the pre–COVID-19 period for loss of a partner (HR, 1.46 [95% CI, 1.41-1.51] vs 1.30 [95% CI, 1.26-1.35]; *P* for difference < .001) and sibling (HR, 1.23 [95% CI, 1.20-1.27] vs 1.16 [95% CI, 1.13-1.19]; *P* for difference = .003). No period difference was noted for the loss of a child or parent. The risk increment after loss of a partner or parent increased with age, while the risk after loss of a child or sibling decreased with age.

**Conclusions and Relevance:**

In this cohort study, bereavement was associated with increased CVD risk both before and during the COVID-19 pandemic; however, a stronger association for partner or sibling loss was noted during the pandemic. These findings suggest that bereavement may be a period of heightened cardiovascular vulnerability, underscoring the importance of targeted clinical monitoring and preventive care for bereaved individuals.

## Key Points

**Question:**

Did the COVID-19 pandemic modify the established association between bereavement and cardiovascular disease (CVD)?

**Findings:**

In this Swedish nationwide cohort of individuals aged 30 years or older during the pre–COVID-19 (2018-2019; n = 5 365 829) and COVID-19 (2020-2021; n = 5 522 898) periods, bereavement was associated with increased risk of myocardial infarction, stroke, heart failure, and other CVD events. Associations were stronger during the COVID-19 period, especially after loss of a partner or sibling, with distinct age-specific patterns by relationship type.

**Meaning:**

These findings suggest that bereavement represents a high-risk period for developing CVD, with risks amplified during the COVID-19 pandemic, underscoring the need for targeted clinical attention.

## Teaser

This cohort study assesses whether the COVID-19 pandemic modified the association between bereavement and cardiovascular disease risk among adults in Sweden.

## Introduction

Bereavement, the experience of losing a close family member or significant person, is a severely stressful life event that nearly all individuals encounter at some point in their lives.^1^ In addition to grief, bereaved individuals often face disruptions in daily routines, financial strain, and a range of physical and psychological health consequences.^1,2,3^

Cardiovascular disease (CVD) is the leading cause of morbidity and mortality worldwide and has been increasingly studied in relation to bereavement. For instance, our previous studies have explored the association of bereavement with both risk and prognosis of CVD, considering timing of loss (eg, childhood, adolescence, young adulthood, middle age, older age, or the perinatal period), type of loss or relationship to the deceased (child, parent, sibling, or partner), cause of death (natural or unnatural), and subtypes of CVD (eg, ischemic heart disease, stroke, atrial fibrillation, and heart failure).^4,5,6,7^ Findings have consistently shown that bereavement is associated with increased risks of incident CVD as well as with worse prognosis of CVD.^8,9,10,11,12^

The COVID-19 pandemic is likely to have introduced additional challenges to the experience of bereavement. Beyond the increased number of deaths, social distancing measures often disrupted traditional mourning practices, limited social support, and delayed access to grief counseling or psychiatric care—all of which may have exacerbated the emotional and broader health consequences of grief.^13,14^ Additionally, the pandemic disrupted health care systems, reducing access to routine care and potentially also screenings for CVD, which may have contributed to undiagnosed or unmanaged CVD risk factors. As a result, the pandemic might have influenced both the experience of bereavement and its resultant grief and the cardiovascular health of the bereaved individuals.^15,16^ However, to date, no study has explored whether the association between bereavement and risk of CVD was modified by the COVID-19 pandemic. To address these knowledge gaps, we conducted a nationwide cohort study to compare the association between loss of a close family member and the risk of incident CVD before and during the COVID-19 pandemic.

## Methods

### Study Population and Follow-Up

This cohort study was conducted as part of the SCIFI-PEARL (Swedish COVID-19 Investigation for Future Insights—A Population Epidemiology Approach Using Register Linkage) project, which is a nationwide, multiregister research project that initially included individuals diagnosed with COVID-19 plus a general population sample^17^ but subsequently expanded to the entire Swedish population. In this study, we included all Swedish residents aged 30 years or older who were alive at the beginning of either of the 2 study periods: the pre–COVID-19 period (January 1, 2018, to December 31, 2019) and the COVID-19 period (January 1, 2020, to December 31, 2021). Individuals were excluded if they had a diagnosis of CVD or had any migration during the 3 years preceding the start of each study period (since data were only available from 2015). To evaluate the association by type of loss, we further constructed 4 restricted cohorts (eFigure 1 in Supplement 1). The cohort of partner loss included individuals with a living partner at baseline, ensuring they were at risk of such exposure. Similarly, we created cohorts for child, parent, and sibling loss. In each cohort, study participants must have at least 1 living close family member in the respective category at baseline. Each individual was followed from baseline until the first occurrence of CVD, emigration, death, or the end of the study period, whichever came first. We followed the Strengthening the Reporting of Observational Studies in Epidemiology (STROBE) reporting guidelines for cohort studies. The study was approved by the Swedish Ethical Review Authority. The boards do not request informed consent for register-based studies.

### Exposure

Bereavement exposure was defined as the death of a partner (married or cohabiting), biological child, parent, or full sibling, within the respective restricted cohorts. Partners were identified via the Swedish Total Population Register^18^ and other relatives via the Swedish Multi-Generation Register.^19^ Information on the date and cause of death was obtained from the Swedish Cause of Death Register.^20^ Exposure was treated as a time-varying variable, meaning that participants contributed follow-up time to the unexposed group until the date of bereavement, and to the exposed group thereafter. If multiple family deaths occurred during the study period, only the first of the relevant type was considered as exposure.

Bereavement was further categorized by the deceased’s underlying cause of death: unnatural cause, CVD, COVID-19 (COVID-19 period only), or other natural causes. Causes of death were classified according to the *International Classification of Diseases and Related Health Problems, Tenth Revision *(*ICD-10*) codes (eTable 1 in Supplement 1).

### Outcomes

The primary outcome of interest was the first diagnosis of any CVD event, identified from an outpatient hospital visit or hospitalization via the Swedish Patient Register^21^ (including both primary and secondary diagnoses) or death via the Swedish Cause of Death Register (using the underlying cause of death), according to the *ICD-10* codes listed in eTable 1 in Supplement 1. In addition to any CVD, we further studied 3 common and severe specific CVD outcomes, ie, myocardial infarction, cerebrovascular disease, and heart failure. In addition, we considered 2 additional secondary CVD outcomes: (1) acute CVD event, defined as a primary diagnosis of CVD recorded at an unplanned or emergency hospital visit according to the Swedish Patient Register; and (2) fatal CVD event, defined as a death within 28 days after the hospital visit for CVD, or a CVD death recorded in the Swedish Cause of Death Register.

### Covariates

We obtained information on age at the start of each study period, sex, highest educational attainment, disposable household income, diagnosis of COVID-19 during the follow-up period, and histories of previous bereavement, diabetes, and psychiatric disorders, as described in the eAppendix in Supplement 1.

### Statistical Analyses

All analyses were conducted separately for each of the 4 restricted cohorts across the 2 study periods. Hazard ratios (HRs) and 95% CIs for any CVD and specific CVD outcomes (including myocardial infarction, cerebrovascular disease, heart failure, acute CVD events and fatal CVD events) in relation to bereavement were estimated using Cox proportional hazards models. Calendar time was selected as the underlying time scale to align risk sets with the onset and progression of the COVID-19 pandemic and to account for contemporaneous changes in health care access and societal conditions. Models were adjusted for age, sex, highest educational attainment, level of disposable household income, and history of diabetes and psychiatric disorders. The proportional hazards assumption was assessed using scaled Schoenfeld residuals; we found no evidence of any major violation of this assumption. A *z* test was conducted to assess whether the HRs differed significantly across the 2 study periods or the 4 subcohorts. To formally assess effect modification by period, we additionally fitted a pooled Cox model including a bereavement × period interaction term with cluster-robust variance estimators.

To explore variations in the association between bereavement and any CVD by time since loss, we used flexible parametric survival models. Additionally, we estimate average HRs during different time intervals (0-7, 8-30, 31-90, 91-365, or >365 days) after the date of loss.

We further examined whether the association differed by the cause of death of the deceased. This analysis served 2 purposes: first, to help disentangle the potential impact of familial confounding (ie, if an effect for CVD-related death might indicate shared genetic or nongenetic risk factors for CVD between family members), and second, to explore whether a death due to unnatural causes^22^ or due to COVID-19^23^ was more strongly associated with CVD risk than a death due to natural causes.

We evaluated effect modification by age using stratified analyses (30-49, 50-69, and ≥70 years) and by formally testing an interaction term between bereavement and age in the Cox model. We also performed stratified analyses based on sex, COVID-19 diagnosis (for the COVID-19 period only), and previous bereavement experience.

We conducted sensitivity analyses to evaluate the robustness of our findings. First, for individuals who experienced more than 1 loss during the study period, follow-up was censored at the time of the second loss. Second, we redefined overall CVD by including only primary inpatient diagnoses or underlying cause of death. Third, for child loss and sibling loss analyses, we tested whether the association with CVD risk differed by the individual’s total number of children or siblings.

We conducted statistical analyses using SAS, version 9.4 (SAS Institute) and R, version 4.4.2 (R Project for Statistical Computing). Statistical significance was defined as a 2-sided *P* < .05. Analyses were performed between September 2024 and August 2025.

## Results

We included a total of 5 365 829 study participants during the pre–COVID-19 period (51.4% female; 48.6% male; median [IQR] age, 51.6 [40.4-64.6] years) and 5 522 898 study participants during the COVID-19 period (51.4% female; 48.6% male, median [IQR] age, 49.8 [38.2-62.8] years). The proportion of bereaved participants was higher during the COVID-19 period than during the pre–COVID-19 period for loss of a partner (1.14% vs 1.10%), loss of a parent (8.10% vs 7.90%), and loss of a sibling (2.16% vs 1.97%). Bereaved participants were more likely to be older and women, to have lower educational attainment and disposable household income, and to have had COVID-19 themselves (for the COVID-19 period only), compared with those in the unexposed group (eTables 2-5 in Supplement 1). These differences were largely consistent between the pre–COVID-19 and COVID-19 periods.

During the pre–COVID-19 period, 372 477 incident CVD cases (6.94%) were identified, compared with 368 902 CVD cases (6.68%) during the COVID-19 period. For both study periods, the crude incidence rate of any CVD was consistently higher among bereaved participants than in the unexposed group (Table 1). The adjusted HRs for CVD risk were 1.30 (95% CI, 1.26-1.35) in the pre–COVID-19 period and 1.46 (95% CI, 1.41-1.51) in the COVID-19 period, respectively, for loss of a partner; 1.25 (95% CI, 1.18-1.33) and 1.28 (95% CI, 1.21-1.36), respectively, for loss of a child; 1.32 (95% CI, 1.29-1.35) and 1.34 (95% CI, 1.30-1.37), respectively, for loss of a parent; and 1.16 (95% CI, 1.13-1.19) and 1.23 (95% CI, 1.20-1.27), respectively, for loss of a sibling. When comparing the pre–COVID-19 and COVID-19 periods using the *z* test, we found higher estimates for partner and sibling loss during the COVID-19 period. However, the formal interaction analysis indicated a statistically significant effect modification by period only for partner loss (*P* for interaction = .003), whereas the interaction was not significant for loss of a sibling, child, or parent.

**Table 1.** Incidence Rates (IRs) and Hazard Ratios (HRs) for Any Cardiovascular Disease Associated With Bereavement Among Individuals Aged 30 Years or Older in Sweden During the Pre–COVID-19 (2018-2019) and COVID-19 (2020-2021) Periods

Bereavement category	Pre–COVID-19 period			COVID-19 period			*P* value for difference between pre–COVID-19 and COVID-19 period
	No. of cases/IR^a^	Crude HR (95% CI)	Adjusted HR (95% CI)^b^	No. of cases/IR^a^	Crude HR (95% CI)	Adjusted HR (95% CI)^b^	
**Cohort of partner loss (pre**–**COVID-19 vs COVID-19 period: 3 190 747 vs 3 262 204)^c^**							
No loss	181 529/31.0	1 [Reference]	1 [Reference]	177 248/29.6	1 [Reference]	1 [Reference]	<.001
Partner loss	3630/108.1	3.46 (3.35-3.57)	1.30 (1.26-1.35)	3900/110.0	3.76 (3.64-3.88)	1.46 (1.41-1.51)	
**Cohort of child loss (pre**–**COVID-19 vs COVID-19 period: 4 440 584 vs 4 537 446)^d^**							
No loss	296 025/36.2	1 [Reference]	1 [Reference]	289 496/34.6	1 [Reference]	1 [Reference]	.59
Child loss	1062/100.8	2.75 (2.59-2.92)	1.25 (1.18-1.33)	1025/97.6	2.84 (2.67-3.02)	1.28 (1.21-1.36)	
**Cohort of parent loss (pre**–**COVID-19 vs COVID-19 period: 3 136 663 vs 3 237 793)^e^**							
No loss	74 497/13.8	1 [Reference]	1 [Reference]	75 609/13.6	1 [Reference]	1 [Reference]	.43
Parent loss	7168/28.4	2.09 (2.04-2.14)	1.32 (1.29-1.35)	7422/27.7	2.10 (2.05-2.16)	1.34 (1.31-1.37)	
**Cohort of sibling loss (pre**–**COVID-19 vs COVID-19 period: 3 579 961 vs 3 694 541)^f^**							
No loss	187 428/28.9	1 [Reference]	1 [Reference]	187 972/28.1	1 [Reference]	1 [Reference]	.003
Sibling loss	5152/74.4	2.57 (2.50-2.64)	1.16 (1.13-1.19)	6173/78.5	2.85 (2.77-2.92)	1.23 (1.20-1.27)	

^a^ IR of any CVD per 1000 person-years.

^b^ Analyses were adjusted for age, sex, highest educational attainment, disposable household income, and history of diabetes and psychiatric disorders.

^c^ The cohort of partner loss included individuals who lost a partner and individuals with no loss of any close family member, provided that they all had at least 1 live partner at baseline.

^d^ The cohort of child loss included individuals who lost a child and individuals with no loss of any close family member, provided that they all had at least 1 live child at baseline.

^e^ The cohort of parent loss included individuals who lost a parent and individuals with no loss of any close family member, provided that they all had at least 1 live parent at baseline.

^f^ The cohort of sibling loss included individuals who lost a sibling and individuals with no loss of any close family member, provided that they all had at least 1 live sibling at baseline.

**Figure 1.**
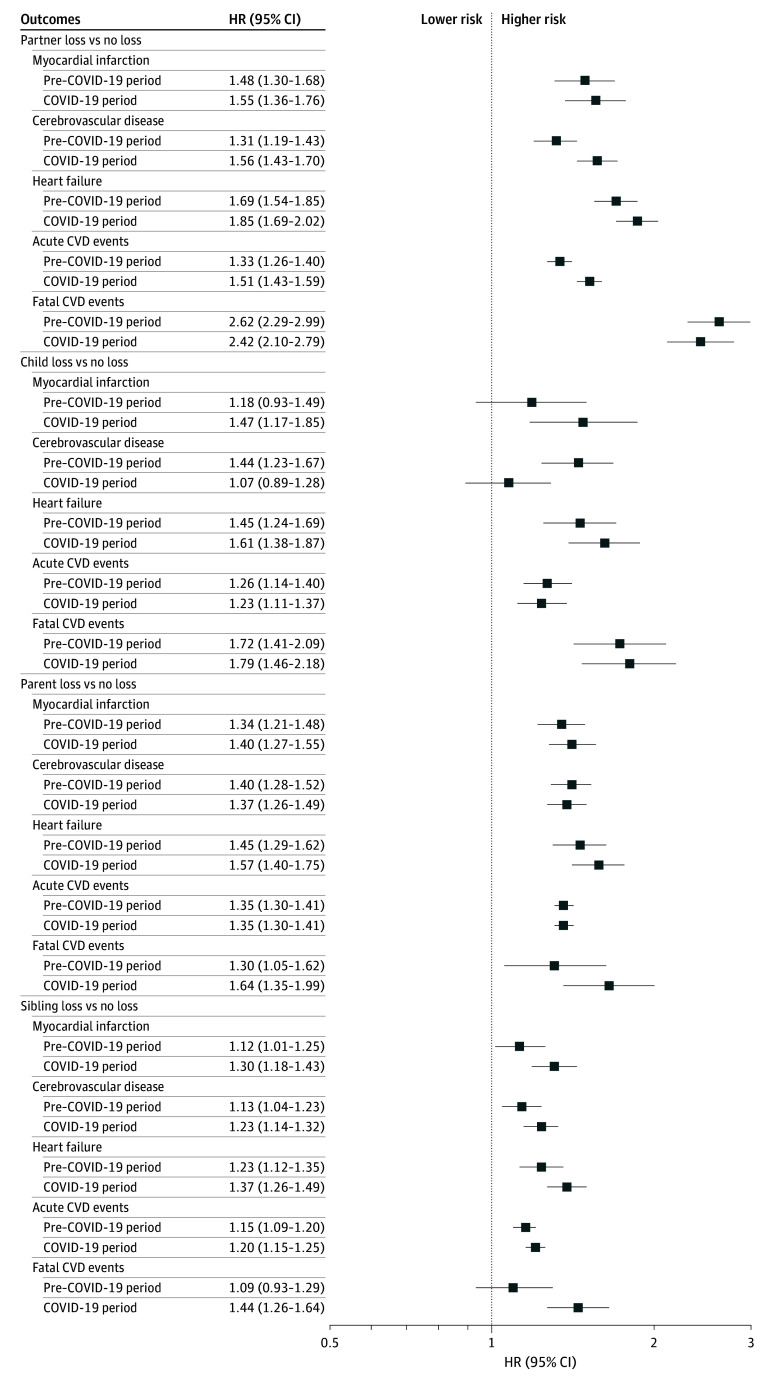
Forest Plot of Hazard Ratios (HRs) for Cardiovascular Disease (CVD) Subtypes After Bereavement During the Pre–COVID-19 and COVID-19 Periods Analyses were adjusted for age, sex, highest educational attainment, disposable household income, and history of diabetes and psychiatric disorders. Statistically significant differences in the associations between the pre–COVID-19 and COVID-19 periods were observed in the following analyses: for partner loss, the *P* values for difference were .006 for cerebrovascular disease and <.001 for acute CVD events; for child loss, the *P* value was .01 for cerebrovascular disease; and for sibling loss, the *P* values were .02 for acute CVD events and .01 for fatal CVD events. No statistically significant differences were found in the remaining comparisons (*P* > .05). Error bars indicate 95% CIs. HRs higher than 1 indicate higher risk of any CVD compared with individuals who did not experience bereavement.

An increased risk was consistently noted for myocardial infarction, cerebrovascular disease, heart failure, and acute or fatal CVD events, in association with bereavement (Figure 1). Compared with other types of loss, loss of a partner in both the pre–COVID-19 and COVID-19 period was associated with the highest risk of fatal CVD events. In addition, we observed that the associations between partner loss and the risk of cerebrovascular disease or acute CVD events, as well as between sibling loss and the risk of acute or fatal CVD events, were stronger during the COVID-19 period than during the pre–COVID-19 period.

**Figure 2.**
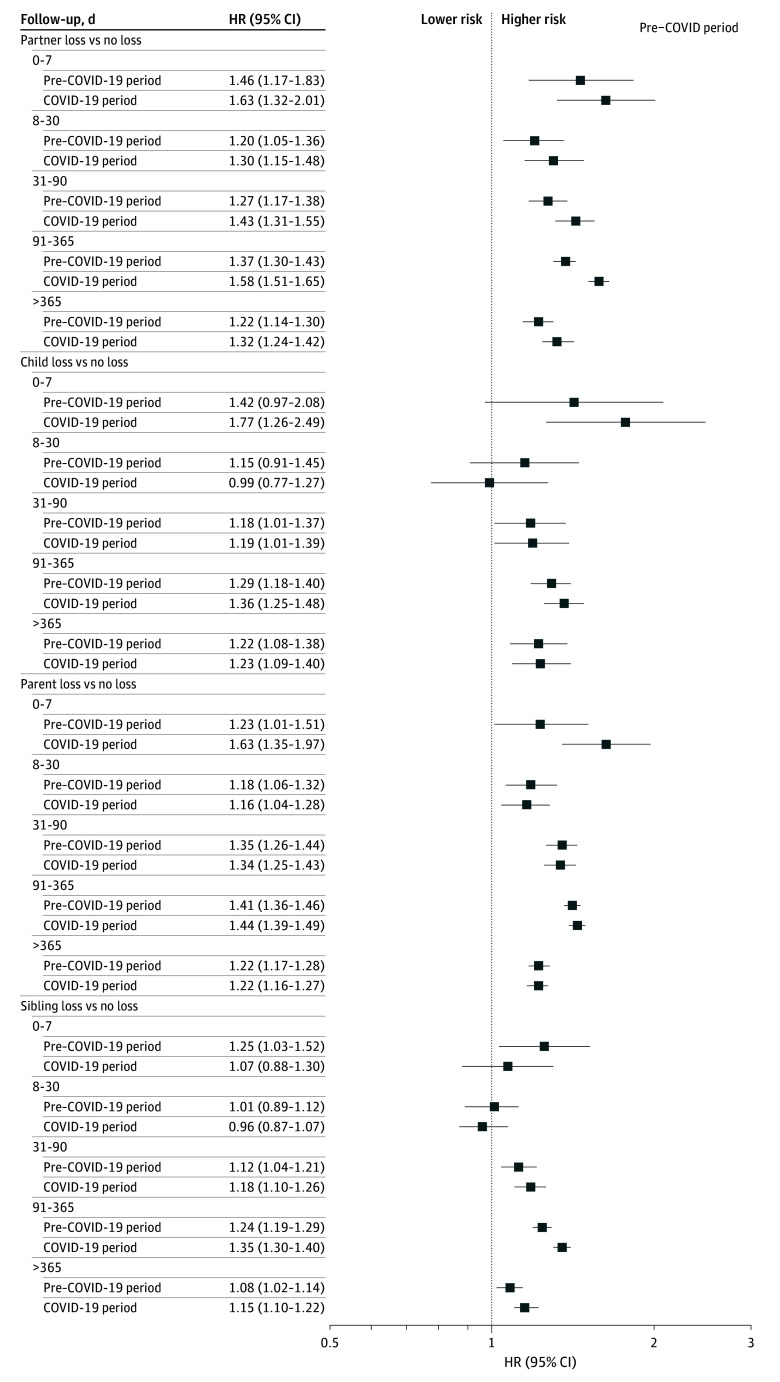
Forest Plot of Hazard Ratios (HRs) for Any Cardiovascular Disease After Bereavement Stratified by Time Since Loss Analyses were adjusted for age, sex, highest educational attainment, disposable household income, and history of diabetes and psychiatric disorders. Statistically significant differences in the associations between the pre–COVID-19 and COVID-19 periods were observed in the following analyses: for partner loss, the *P* value for difference was < .001 for the period of 91 to 365 days; for parent loss, the *P* value for difference was .05 for the period of 0 to 7 days; and for sibling loss, the *P* value for difference was .002 for the period of 91 to 365 days. No statistically significant differences were found in the remaining comparisons (*P* > .05). Error bars indicate 95% CIs. HRs higher than 1 indicate higher risk of any CVD compared with individuals not experiencing bereavement.

Flexible parametric models showed that the risk of any CVD after bereavement was generally highest during the first 90 days after loss and decreased gradually afterwards (eFigures 2-5 in Supplement 1). When the follow-up period was divided into specific intervals, we observed a similar pattern for both the pre–COVID-19 and COVID-19 periods: the highest risk was mostly noted during the first 7 days after the loss (Figure 2).

**Figure 3.**
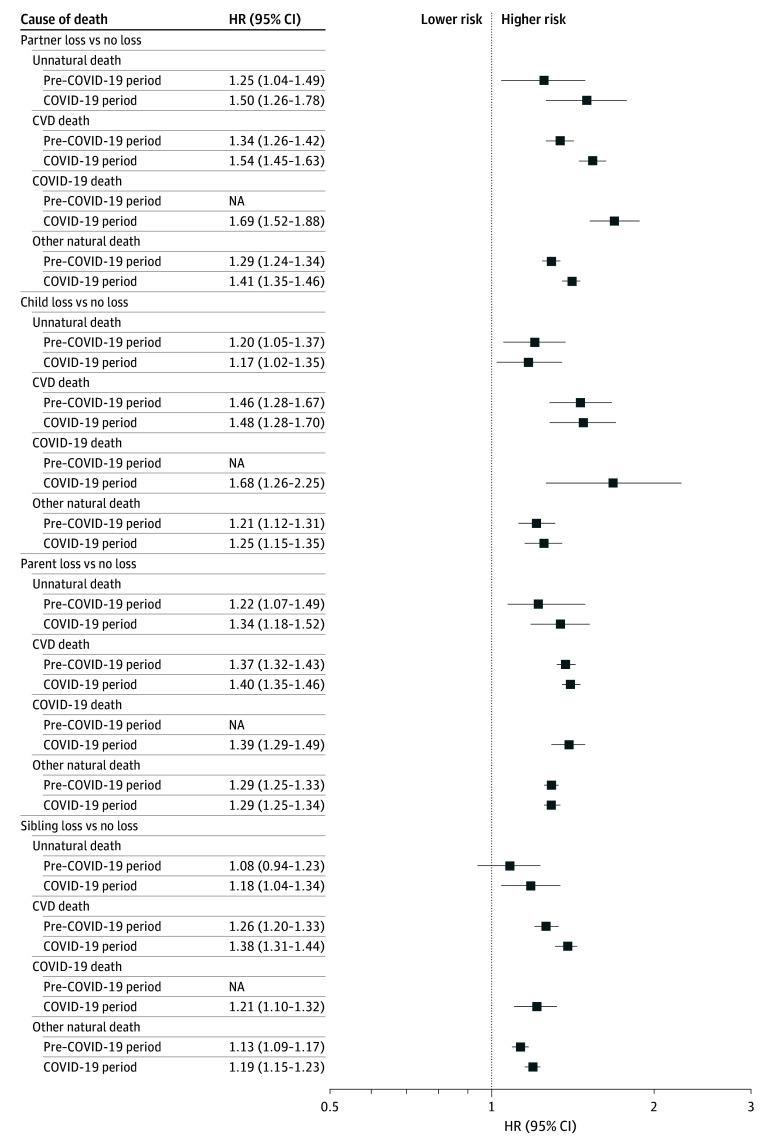
Forest Plot of Hazard Ratios (HRs) for Any Cardiovascular Disease (CVD) After Bereavement Stratified by Cause of Death Analyses were adjusted for study participants’ age, sex, highest educational attainment, disposable household income, and history of diabetes and psychiatric disorders. Statistically significant differences in the associations between the pre–COVID-19 and COVID-19 periods were observed in the following analyses: for partner loss, the *P* values for difference were .002 for CVD death and .007 for other natural death; and for sibling loss, the *P* values for difference were .01 for CVD death and .04 for other natural death. No statistically significant differences were found in the remaining comparisons (*P* > .05). Error bars indicate 95% CIs. HRs higher than 1 indicate higher risk of any CVD compared with individuals who did not experience bereavement.

When we examined the association by the cause of death of the deceased, we found that death due to CVD was more strongly associated with CVD risk in the bereaved individuals (Figure 3), although other causes also showed elevated risk. Notably, during the COVID-19 period, individuals who lost a partner or child due to COVID-19 infection had the highest CVD risk compared with those who were bereaved due to other causes, although the corresponding 95% CIs overlapped. When comparing the 2 study periods, partner loss and sibling loss due to CVD-related or other natural causes were more strongly associated with CVD risk during the COVID-19 period than the pre–COVID-19 period.

The magnitude of the association between bereavement and increased CVD risk varied by age and type of loss (Table 2). For both partner loss and parent loss, the risk of CVD increased with age at loss. In contrast, child loss and sibling loss were associated with decreasing CVD risk as age increased. The association between partner or sibling loss and CVD was significantly stronger during the COVID-19 period than the pre–COVID-19 period among individuals aged 70 years or older.

**Table 2.** Incidence Rates (IRs) and Hazard Ratios (HRs) for Any Cardiovascular Disease Associated With Bereavement During the Pre–COVID-19 (2018-2019) and COVID-19 (2020-2021) Periods, Stratified Analysis by Age at Cohort Entry

Age group	Pre–COVID-19 period			COVID-19 period			*P* value for difference between pre–COVID-19 and COVID-19 period
	No. of cases/IR^a^	Crude HR (95% CI)	Adjusted HR (95% CI)^b^	No. of cases/IR^a^	Crude HR (95% CI)	Adjusted HR (95% CI)^b^	
**Cohort of partner loss (pre–COVID-19 vs COVID-19 period: 3 190 747 vs 3 262 204)**							
30-49 y (Pre–COVID-19 vs COVID-19 period: 1 398 936 vs 1 428 589)							
No loss	23 267/8.6	1 [Reference]	1 [Reference]	23 530/8.5	1 [Reference]	1 [Reference]	.78
Partner loss	19/12.0	1.42 (0.90-2.22)	1.19 (0.76-1.86)	21/13.1	1.59 (1.04-2.43)	1.30 (0.85-2.00)	
50-69 y (Pre–COVID-19 vs COVID-19 period: 1 242 055 vs 1 251 913)							
No loss	71 737/31.8	1 [Reference]	1 [Reference]	68 929/30.3	1 [Reference]	1 [Reference]	.41
Partner loss	432/47.1	1.50 (1.36-1.65)	1.21 (1.10-1.33)	410/44.0	1.49 (1.35-1.64)	1.28 (1.16-1.41)	
≥70 y (Pre–COVID -19 vs COVID-19 period: 549 756 vs 581 702)							
No loss	86 525/96.8	1 [Reference]	1 [Reference]	84 789/89.3	1 [Reference]	1 [Reference]	<.001
Partner loss	3179/139.2	1.44 (1.39-1.49)	1.23 (1.19-1.27)	3469/141.4	1.63 (1.57-1.68)	1.40 (1.35-1.45)	
*P* for interaction	.04			.90			
**Cohort of child loss (pre**–**COVID-19 vs COVID-19 period: 4 440 584 vs 4 537 446)**							
30-49 y (Pre–COVID-19 vs COVID-19 period: 1 848 522 vs 1 876 231)							
No loss	30 992/8.7	1 [Reference]	1 [Reference]	31 218/8.6	1 [Reference]	1 [Reference]	.87
Child loss	24/15.7	1.84 (1.23-2.74)	1.57 (1.05-2.34)	22/14.9	1.78 (1.17-2.70)	1.65 (1.08-2.50)	
50-69 y (Pre–COVID-19 vs COVID-19 period: 1 725 752 vs 1 747 225)							
No loss	103 836/33.0	1 [Reference]	1 [Reference]	100 501/31.5	1 [Reference]	1 [Reference]	.34
Child loss	152/41.9	1.28 (1.10-1.51)	1.13 (0.97-1.33)	149/43.7	1.42 (1.21-1.67)	1.26 (1.08-1.48)	
≥70 y (Pre–COVID-19 vs COVID-19 period: 866 310 vs 913 990)							
No loss	161 197/111.3	1 [Reference]	1 [Reference]	157 777/102.6	1 [Reference]	1 [Reference]	.87
Child loss	886/164.9	1.48 (1.38-1.58)	1.18 (1.11-1.26)	854/152.0	1.52 (1.42-1.62)	1.19 (1.11-1.27)	
*P* or interaction	.58			.82			
**Cohort of parent loss (pre**–**COVID-19 vs COVID-19 period: 3 136 663 vs 3 237 793)**							
30-49 y (Pre–COVID-19 vs COVID-19 period: 1 956 587 vs 2 009 631)							
No loss	31 010/8.6	1 [Reference]	1 [Reference]	31 220/8.4	1 [Reference]	1 [Reference]	.11
Parent loss	907/11.5	1.35 (1.27-1.45)	1.15 (1.08-1.23)	995/12.0	1.48 (1.39-1.57)	1.24 (1.17-1.33)	
50-69 y (Pre–COVID-19 vs COVID-19 period: 1 139 241 vs 1 183 534)							
No loss	41 400/23.8	1 [Reference]	1 [Reference]	42 263/23.5	1 [Reference]	1 [Reference]	.46
Parent loss	5381/33.5	1.45 (1.40-1.49)	1.27 (1.23-1.31)	5446/31.8	1.41 (1.37-1.45)	1.25 (1.22-1.29)	
≥70 y (Pre–COVID-19 vs COVID-19 period: 40 835 vs 44 628)							
No loss	2087/50.1	1 [Reference]	1 [Reference]	2126/46.5	1 [Reference]	1 [Reference]	.08
Parent loss	880/70.1	1.48 (1.37-1.60)	1.46 (1.35-1.58)	981/71.3	1.64 (1.52-1.77)	1.61 (1.49-1.74)	
*P* for interaction	<.001			<.001			
**Cohort of sibling loss (pre**–**COVID-19 vs COVID-19 period: 3 579 961 vs 3 694 541)**							
30-49 y (Pre–COVID-19 vs COVID-19 period:1 585 219 vs 1 626 255)							
No loss	26 233/8.6	1 [Reference]	1 [Reference]	26 123/8.3	1 [Reference]	1 [Reference]	.05
Sibling loss	83/17.6	2.07 (1.67-2.57)	1.71 (1.37-2.12)	54/11.8	1.46 (1.12-1.91)	1.22 (0.94-1.60)	
50-69 y (Pre–COVID-19 vs COVID-19 period: 1 449 161 vs 1 454 414)							
No loss	82 590/32.2	1 [Reference]	1 [Reference]	79 094/30.7	1 [Reference]	1 [Reference]	<.001
Sibling loss	1441/47.6	1.50 (1.42-1.58)	1.16 (1.10-1.22)	1503/50.1	1.68 (1.60-1.77)	1.32 (1.26-1.39)	
≥70 y (Pre–COVID-19 vs COVID-19 period: 545 581 vs 613 872)							
No loss	78 605/90.5	1 [Reference]	1 [Reference]	82 755/85.4	1 [Reference]	1 [Reference]	.02
Sibling loss	3628/105.9	1.48 (1.38-1.58)	1.12 (1.08-1.15)	4616/104.9	1.27 (1.23-1.31)	1.18 (1.15-1.22)	
*P* for interaction	<.001			<.001			

^a^ IR of any CVD per 1000 person-years.

^b^ Analyses were adjusted for age, sex, highest educational attainment, disposable household income, and history of diabetes and psychiatric disorders.

The association between bereavement and CVD risk did not differ significantly between men and women, by COVID-19 status, or by bereavement history (eTable 6-8 in Supplement 1). The results remained largely unchanged when restricting the exposed group to individuals who experienced only 1 loss during the follow-up period, as well as when CVD cases were more strictly defined (eTable 9 in Supplement 1). The number of children or siblings in the family did not substantially alter the associations between child or sibling loss and CVD risk (eTables 10 and 11 in Supplement 1).

## Discussion

In this cohort study, death of a partner, child, parent, or sibling was associated with an increased risk of overall and severe CVD outcomes both before and during the COVID-19 pandemic. Although estimates were generally higher during the COVID-19 period, particularly for partner and sibling loss, the formal interaction analysis indicated a statistically significant period difference only for partner loss. The association appeared to increase with age after partner or parent loss but to decrease with age after child or sibling loss.

We hypothesized that the impact of bereavement on CVD might be amplified during the pandemic. The present study did reveal stronger associations during COVID-19 for partner loss, and to a lesser extent sibling loss, but not for parent or child loss. One plausible explanation is that partner and sibling loss often removes key sources of emotional and practical daily support, and pandemic-related social restrictions may have intensified this disruption. We also found that bereavement due to a COVID-19–related death, particularly after the loss of a partner or a child, was associated with a higher risk of CVD compared with bereavement from other natural causes. This elevated risk may be attributable to the more intense and acute grief triggered by the unexpected nature of COVID-19–related deaths. This explanation is supported by a Dutch study^23^ showing more severe grief after COVID-19–related bereavement than after other natural losses.

Although we anticipated that partner or child loss would have a stronger impact on CVD risk compared with parent or sibling loss, no clear differences were observed. Because each bereavement type was evaluated within a separate restricted cohort with distinct cohort composition, age structure, and baseline CVD risk, HRs across loss types are not directly comparable. Notably, the association increased with age after loss of a partner or parent, but decreased with age after loss of a child or sibling. To our knowledge, this age-specific pattern has not been previously reported. This pattern may reflect differences in the psychological, social, and physiological consequences of bereavement depending on both the type of relationship and the life stage at which the loss occurs. For instance, older individuals may be more emotionally and practically dependent on a partner, and the loss of a partner may lead to profound loneliness, disrupted routines, and reduced support—factors that can heighten stress and age-related CVD risk. In contrast, the emotional and physiological impact of losing a child or sibling may be more intense for younger or middle-aged individuals, particularly when they still have caregiving responsibilities. In addition, the heterogeneous structure of the 4 restricted cohorts, involving differences in baseline CVD incidence and the burden of competing events (non-CVD death) across age groups, may also contribute to the different age-specific patterns. For example, the child-loss cohort exhibited both higher baseline CVD incidence and greater non-CVD mortality (data not shown) at older ages, both of which are factors that could attenuate relative risk estimates in the oldest age group. Taken together, these findings suggest that the impact of bereavement on CVD risk is determined not only by the relationship closeness but also by the timing of loss, underscoring the importance of considering age-specific dynamics in future studies.

### Limitations

Some limitations should be acknowledged. First, there is a potential for underreporting of CVD events. Although the Swedish Patient Register has high validity for most CVD diagnoses, such as myocardial infraction and heart failure,^24,25^ mild CVD might not have been captured as this register does not include information on primary care. Additionally, the pandemic resulted in a substantial decline in hospital admission for CVD and longer delays between symptom onset and medical treatment,^26^ which could likely contribute to some underreporting of incident CVD cases during the pandemic period. If such underreporting occurred similarly in bereaved and nonbereaved individuals, the associations during the pandemic would likely be biased toward the null, potentially exaggerating the contrast with the pre–COVID-19 period. However, if underdiagnosis during the pandemic differed substantially by bereavement status, some differential misclassification cannot be ruled out; in that case, the direction and magnitude of the resulting bias, including its impact on period-specific differences, would remain uncertain. Second, we lacked data on key CVD risk factors (eg, smoking, obesity) and therefore could not adjust for them in our analyses. Although these factors mainly influence individual CVD risk and are unlikely to strongly influence bereavement, residual confounding remains possible, particularly when the deceased died from CVD-related causes. In addition, unmeasured shared genetic or environmental risk factors within families may partly contribute to the observed associations, as suggested by the stronger associations after a CVD-related loss. Third, deaths due to causes other than CVD represent competing events, and censoring of these events may be informative rather than independent, which affects interpretation of the estimated HRs.^27^ Fourth, given the unique Swedish context, including its universal welfare system and less invasive mitigating strategies during the pandemic,^28,29^ the generalizability of our findings to other settings remains unknown.

## Conclusion

In this cohort study, death of a close family member was associated with an increased risk of CVD. The risk increment after loss of a partner or a sibling was stronger during the COVID-19 period compared to the pre–COVID-19 period, suggesting a potential interaction between bereavement and psychosocial conditions during the COVID-19 pandemic. These findings demonstrate the compounded cardiovascular burden among bereaved individuals during the COVID-19 pandemic and underscore the need for targeted prevention and support to reduce CVD risk in this vulnerable population, particularly during major health crises or other stressful circumstances.

## Notes

Supplement 1. **eTable 1.** *International Classification of Diseases* Codes Used to Define Causes of Death and Medical Conditions Relevant for the Study **eTable 2. **Baseline Characteristics of Participants in the Cohort of Partner Loss During the Pre-COVID and COVID Periods **eTable 3. **Baseline Characteristics of Participants in the Cohort of Child Loss During the Pre-COVID and COVID Periods **eTable 4. **Baseline Characteristics of Participants in the Cohort of Parent Loss During the Pre-COVID and COVID Periods **eTable 5. **Baseline Characteristics of Participants in the Cohort of Sibling Loss During the Pre-COVID and COVID Periods **eTable 6. **Incidence Rate (Per 1000 Person-Years) and Hazard Ratio of Any Cardiovascular Disease According to Bereavement During the Pre-COVID and COVID Periods, Stratified Analyses by Sex **eTable 7. **Incidence Rate (Per 1000 Person-Years) and Hazard Ratio of Any Cardiovascular Disease According to Bereavement During the COVID Period, Stratified Analyses by COVID-19 Diagnosis **eTable 8. **Incidence Rate (Per 1000 Person-Years) and Hazard Ratio of Any Cardiovascular Disease According to Bereavement During the Pre-COVID And COVID Periods, Stratified Analyses By Bereavement History **eTable 9. **Sensitivity Analyses for the Association Between Bereavement and the Risk of Any Cardiovascular Disease **eTable 10.** Incidence Rate (Per 1000 Person-Years) and Hazard Ratio of Any Cardiovascular Disease According to Child Loss During the Pre-COVID and COVID Periods, Stratified Analyses by Number of Children in the Family **eTable 11.** Incidence Rate (Per 1000 Person-Years) and Hazard Ratio of Any Cardiovascular Disease According to Sibling Loss During the Pre-COVID and COVID Periods, Stratified Analyses by Number of Siblings in the Family **eFigure 1. **Study Design **eFigure 2. **Hazard Ratios of Any Cardiovascular Disease in Relation to Partner Loss, Analyses Using Flexible Parametric Survival Models **eFigure 3. **Hazard Ratios of Any Cardiovascular Disease in Relation to Child Loss, Analyses Using Flexible Parametric Survival Models **eFigure 4. **Hazard Ratios of Any Cardiovascular Disease in Relation to Parent Loss, Analyses Using Flexible Parametric Survival Models **eFigure 5. **Hazard Ratios of Any Cardiovascular Disease in Relation to Sibling Loss, Analyses Using Flexible Parametric Survival Models **eAppendix. **Description of the Source, the Measurement and the Categorization of Covariates
eReferences

Supplement 2.
Data Sharing Statement
