# Supplementary material for: Bereavement and Risk of Cardiovascular Disease Before and During the COVID-19 Pandemic
Source: JAMA Netw Open. 2026 Apr 24;9(4):e269102. doi: 10.1001/jamanetworkopen.2026.9102 (PMC13109797; doi:10.1001/jamanetworkopen.2026.9102)
Supplement: Supplement 1. — eTable 1. International Classification of Diseases Codes Used to Define Causes of Death and Medical Conditions Relevant for the Study eTable 2. Baseline Characteristics of Participants in the Cohort of Partner Loss During the Pre-COVID and COVID Periods eTable 3. Baseline Characteristics of Participants in the Cohort of Child Loss During the Pre-COVID and COVID Periods eTable 4. Baseline Characteristics of Participants in the Cohort of Parent Loss During the Pre-COVID and COVID Periods eTable 5. Baseline Characteristics of Participants in the Cohort of Sibling Loss During the Pre-COVID and COVID Periods eTable 6. Incidence Rate (Per 1000 Person-Years) and Hazard Ratio of Any Cardiovascular Disease According to Bereavement During the Pre-COVID and COVID Periods, Stratified Analyses by Sex eTable 7. Incidence Rate (Per 1000 Person-Years) and Hazard Ratio of Any Cardiovascular Disease According to Bereavement During the COVID Period, Stratified Analyses by COVID-19 Diagnosis eTable 8. Incidence Rate (Per 1000 Person-Years) and Hazard Ratio of Any Cardiovascular Disease According to Bereavement During the Pre-COVID And COVID Periods, Stratified Analyses By Bereavement History eTable 9. Sensitivity Analyses for the Association Between Bereavement and the Risk of Any Cardiovascular Disease eTable 10. Incidence Rate (Per 1000 Person-Years) and Hazard Ratio of Any Cardiovascular Disease According to Child Loss During the Pre-COVID and COVID Periods, Stratified Analyses by Number of Children in the Family eTable 11. Incidence Rate (Per 1000 Person-Years) and Hazard Ratio of Any Cardiovascular Disease According to Sibling Loss During the Pre-COVID and COVID Periods, Stratified Analyses by Number of Siblings in the Family eFigure 1. Study Design eFigure 2. Hazard Ratios of Any Cardiovascular Disease in Relation to Partner Loss, Analyses Using Flexible Parametric Survival Models eFigure 3. Hazard Ratios of Any Cardiovascular Disease in Relation to Child Loss, Analyses Using Fle [file jamanetwopen-e269102-s001.pdf]

**Supplemental Online Content**

Yang F, Li S, Barker MM, et al. Bereavement and risk of cardiovascular disease before and during the COVID-19 pandemic. *JAMA Newt Open*. 2026;9(4):e269102.
doi:10.1001/jamanetworkopen.2026.9102

**eTable 1.** *International Classification of Diseases* Codes Used to Define Causes of Death and Medical Conditions Relevant for the Study
**eTable 2.** Baseline Characteristics of Participants in the Cohort of Partner Loss During the Pre-COVID and COVID Periods
**eTable 3.** Baseline Characteristics of Participants in the Cohort of Child Loss During the Pre-COVID and COVID Periods
**eTable 4.** Baseline Characteristics of Participants in the Cohort of Parent Loss During the Pre-COVID and COVID Periods
**eTable 5.** Baseline Characteristics of Participants in the Cohort of Sibling Loss During the Pre-COVID and COVID Periods
**eTable 6.** Incidence Rate (Per 1000 Person-Years) and Hazard Ratio of Any Cardiovascular Disease According to Bereavement During the Pre-COVID and COVID Periods, Stratified Analyses by Sex
**eTable 7.** Incidence Rate (Per 1000 Person-Years) and Hazard Ratio of Any Cardiovascular Disease According to Bereavement During the COVID Period, Stratified Analyses by COVID-19 Diagnosis
**eTable 8.** Incidence Rate (Per 1000 Person-Years) and Hazard Ratio of Any Cardiovascular Disease According to Bereavement During the Pre-COVID And COVID Periods, Stratified Analyses By Bereavement History
**eTable 9.** Sensitivity Analyses for the Association Between Bereavement and the Risk of Any Cardiovascular Disease
**eTable 10.** Incidence Rate (Per 1000 Person-Years) and Hazard Ratio of Any Cardiovascular Disease According to Child Loss During the Pre-COVID and COVID Periods, Stratified Analyses by Number of Children in the Family
**eTable 11.** Incidence Rate (Per 1000 Person-Years) and Hazard Ratio of Any Cardiovascular Disease According to Sibling Loss During the Pre-COVID and COVID Periods, Stratified Analyses by Number of Siblings in the Family
**eFigure 1.** Study Design
**eFigure 2.** Hazard Ratios of Any Cardiovascular Disease in Relation to Partner Loss, Analyses Using Flexible Parametric Survival Models
**eFigure 3.** Hazard Ratios of Any Cardiovascular Disease in Relation to Child Loss, Analyses Using Flexible Parametric Survival Models
**eFigure 4.** Hazard Ratios of Any Cardiovascular Disease in Relation to Parent Loss, Analyses Using Flexible Parametric Survival Models
**eFigure 5.** Hazard Ratios of Any Cardiovascular Disease in Relation to Sibling Loss, Analyses Using Flexible Parametric Survival Models
**eAppendix 1.** Description of the Source, the Measurement and the Categorization of Covariates
**eReferences**

This supplemental material has been provided by the authors to give readers additional information about their work.

© 2026 Yang F et al. *JAMA Netw Open.*

**eTable 1. International Classification of Diseases codes used to define causes of death and medical conditions relevant for the study**

| | ICD-10 |
|---|---|
| **Cause of death** | |
| Unnatural death | R95, R96, R98, V01-Y98 |
| Death due to cardiovascular disease | I00-I99 |
| Death due to COVID-19 | U07.1, U07.2, U09.9, U10.9 |
| Other natural death | All the other codes |
| **Cardiovascular disease** | |
| Any cardiovascular disease | I00-I99 |
| Myocardial infarction | I21, I22 |
| Cerebrovascular diseases | I6, G45 |
| Heart failure | I11.0, I13.0, I13.2, I50 |
| **Covariates** | |
| COVID-19 | U07.1, U07.2, U09.9, U10.9 |
| Diabetes | E10-E14 |
| Psychiatric disorders | F00-F99 |

ICD = International Statistical Classification of Diseases

© 2026 Yang F et al. *JAMA Netw Open.*

**eTable 2. Baseline characteristics of Participants in the cohort of partner loss during the pre-COVID and COVID periods**

| Characteristics | Pre-COVID period | | COVID period | |
|---|---|---|---|---|
| | No loss (N= 3,155,626) | Loss of a partner (N= 35,121) | No loss (N=3,225,043) | Loss of a partner (N=37,161) |
| **Age at the start of follow-up, median (IQR), years** | 51.4 (41.3-64.2) | 74.2 (66.7-81.1) | 51.6 (41.0-64.3) | 74.8 (66.9-81.3) |
| **Sex, N (%)** | | | | |
| Male | 1,557,607 (49.4) | 10,760 (30.6) | 1,589,860 (49.3) | 10,998 (29.6) |
| Female | 1,598,019 (50.6) | 24,361 (69.4) | 1,635,183 (50.7) | 26,163 (70.4) |
| **Highest educational attainment, N (%)** | | | | |
| ≤9 years | 437,354 (13.9) | 11,401 (32.5) | 420,181 (13.0) | 11,355 (30.6) |
| 10-12 years | 1,348,803 (42.7) | 14,591 (41.5) | 1,352,612 (41.9) | 15,630 (42.1) |
| >12 years | 1,350,236 (42.8) | 8842 (25.2) | 1,429,719 (44.3) | 9840 (26.5) |
| Unknown | 19,233 (0.6) | 287 (0.8) | 22,531 (0.7) | 336 (0.9) |
| **Disposable household income, N (%)** | | | | |
| Low | 1,037,996 (32.9) | 24,847 (70.7) | 1,060,104 (32.9) | 26,273 (70.7) |
| Middle | 1,056,747 (33.5) | 5977 (17.0) | 1,080,125 (33.5) | 6322 (17.0) |
| High | 1,060,883 (33.6) | 4297 (12.2) | 1,084,814 (33.6) | 4566 (12.3) |
| **History of psychiatric disorders, N (%)** | | | | |
| No | 3,029,306 (96.0) | 33,509 (95.4) | 3,095,706 (96.0) | 35,546 (95.7) |
| Yes | 126,320 (4.0) | 1612 (4.6) | 129,337 (4.0) | 1615 (4.3) |
| **History of diabetes, N (%)** | | | | |
| No | 3,103,832 (98.4) | 33,940 (96.6) | 3,170,850 (98.3) | 35,872 (96.5) |
| Yes | 51,794 (1.6) | 1181 (3.4) | 54,193 (1.7) | 1289 (3.5) |
| **History of bereavement, N (%)** | | | | |
| No | 2,959,855 (93.8) | 32,929 (93.8) | 3,029,674 (93.9) | 34,753 (93.5) |
| Yes | 195,771 (6.2) | 2192 (6.2) | 195,369 (6.1) | 2408 (6.5) |
| **COVID-19 status, N (%)** | | | | |
| No | - | - | 2,313,210 (71.7) | 30,709 (82.6) |
| Yes | - | - | 911,833 (28.3) | 6452 (17.4) |

© 2026 Yang F et al. *JAMA Netw Open.*

**eTable 3. Baseline characteristics of participants in the cohort of child loss during the pre-COVID and COVID periods**

| Characteristics | Pre-COVID period | | COVID period | |
|---|---|---|---|---|
| | No loss (N=4,429,760) | Loss of a child (N=10,824) | No loss (N=4,526,562) | Loss of a child (N=10,884) |
| **Age at the start of follow-up, median (IQR), years** | 52.6 (41.8-65.7) | 70.3 (56.3-80.4) | 52.9 (41.8-65.9) | 71.5 (57.1-80.8) |
| **Sex, N (%)** | | | | |
| Male | 2,049,408 (46.3) | 4038 (37.3) | 2,092,968 (46.2) | 4053 (37.2) |
| Female | 2,380,352 (53.7) | 6786 (62.7) | 2,433,594 (53.8) | 6831 (62.8) |
| **Highest educational attainment, N (%)** | | | | |
| ≤9 years | 708,588 (16.0) | 3603 (33.3) | 684,751 (15.1) | 3493 (32.1) |
| 10-12 years | 1,914,084 (43.2) | 4758 (44.0) | 1,939,019 (42.8) | 4787 (44.0) |
| >12 years | 1,686,148 (38.1) | 2334 (21.6) | 1,797,777 (39.7) | 2464 (22.6) |
| Unknown | 120,940 (2.7) | 129 (1.2) | 105,015 (2.3) | 140 (1.3) |
| **Disposable household income, N (%)** | | | | |
| Low | 1,440,337 (32.5) | 6348 (58.6) | 1,478,346 (32.7) | 6586 (60.5) |
| Middle | 1,442,641 (32.6) | 2520 (23.3) | 1,481,381 (32.7) | 2502 (23.0) |
| High | 1,447,685 (32.7) | 1891 (17.5) | 1,487,067 (32.9) | 1733 (15.9) |
| Unknown | 99,097 (2.2) | 65 (0.6) | 79,768 (1.8) | 63 (0.6) |
| **History of psychiatric disorders, N (%)** | | | | |
| No | 4,181,649 (94.4) | 10,019 (92.6) | 4,273,020 (94.4) | 10,081 (92.6) |
| Yes | 248,111 (5.6) | 805 (7.4) | 253,542 (5.6) | 803 (7.4) |
| **History of diabetes, N (%)** | | | | |
| No | 4,350,476 (98.2) | 10,452 (96.6) | 4,443,317 (98.2) | 10,497 (96.4) |
| Yes | 79,284 (1.8) | 372 (3.4) | 83,245 (1.8) | 387 (3.6) |
| **History of bereavement, N (%)** | | | | |
| No | 4,154,895 (93.8) | 10,086 (93.2) | 4,249,020 (93.9) | 10,188 (93.6) |
| Yes | 274,865 (6.2) | 738 (6.8) | 277,542 (6.1) | 696 (6.4) |
| **COVID-19 status, N (%)** | | | | |
| No | - | - | 3,317,979 (73.3) | 8743 (80.3) |
| Yes | - | - | 1,208,583 (26.7) | 2141 (19.7) |

© 2026 Yang F et al. *JAMA Netw Open.*

**eTable 4. Baseline Characteristics of Participants in the cohort of parent loss during the pre-COVID and COVID periods**

| Characteristics | Pre-COVID period | | COVID period | |
|---|---|---|---|---|
| | No loss (N=2,888,998) | Loss of a parent (N=247,665) | No loss (N=2,975,353) | Loss of a parent (N=262,440) |
| **Age at the start of follow-up, median (IQR), years** | 44.3 (36.4-52.3) | 53.8 (46.6-60.5) | 44.4 (36.0-52.7) | 54.1 (46.7-60.6) |
| **Sex, N (%)** | | | | |
| Male | 1,469,086 (50.9) | 123,740 (50.0) | 1,512,467 (50.8) | 131,278 (50.0) |
| Female | 1,419,912 (49.1) | 123,925 (50.0) | 1,462,886 (49.2) | 131,162 (50.0) |
| **Highest educational attainment, N (%)** | | | | |
| ≤9 years | 247,718 (8.6) | 28,485 (11.5) | 239,335 (8.0) | 27,812 (10.6) |
| 10-12 years | 1,316,116 (45.6) | 119,617 (48.3) | 1,325,270 (44.5) | 125,219 (47.7) |
| >12 years | 1,301,889 (45.1) | 98,059 (39.6) | 1,379,881 (46.4) | 107,476 (41.0) |
| Unknown | 23,275 (0.8) | 1504 (0.6) | 30,867 (1.0) | 1933 (0.7) |
| **Disposable household income, N (%)** | | | | |
| Low | 953,310 (33.0) | 87,601 (35.4) | 979,720 (32.9) | 92,317 (35.2) |
| Middle | 961,607 (33.3) | 78,000 (31.5) | 990,121 (33.3) | 82,099 (31.3) |
| High | 961,833 (33.3) | 81,205 (32.8) | 987,378 (33.2) | 86,864 (33.1) |
| Unknown | 12,248 (0.4) | 859 (0.3) | 18,134 (0.6) | 1160 (0.4) |
| **History of psychiatric disorders, N (%)** | | | | |
| No | 2,674,788 (92.6) | 231,702 (93.6) | 3,378,398 (93.5) | 75,249 (94.4) |
| Yes | 214,210 (7.4) | 15,963 (6.4) | 236,399 (6.5) | 4495 (5.6) |
| **History of diabetes, N (%)** | | | | |
| No | 2,856,693 (98.9) | 244,182 (98.6) | 3,554,838 (98.3) | 77,330 (97.0) |
| Yes | 32,305 (1.1) | 3483 (1.4) | 59,959 (1.7) | 2414 (3.0) |
| **History of bereavement, N (%)** | | | | |
| No | 2,753,482 (95.3) | 233,773 (94.4) | 3,351,116 (92.7) | 71,544 (89.7) |
| Yes | 135,516 (4.7) | 13,892 (5.6) | 263,681 (7.3) | 8200 (10.3) |
| **COVID-19 status, N (%)** | | | | |
| No | - | - | 2,675,435 (74.0) | 68,288 (85.6) |
| Yes | - | - | 939,362 (26.0) | 11,456 (14.4) |

© 2026 Yang F et al. *JAMA Netw Open.*

**eTable 5. Baseline characteristics of participants in the cohort of sibling loss during the pre-COVID and COVID periods**

| Characteristics | Pre-COVID period | | COVID period | |
|---|---|---|---|---|
| | No loss (N= 3,509,455) | Loss of a sibling (N= 70,506) | No loss (N=3,614,797) | Loss of a sibling (N=79,744) |
| **Age at the start of follow-up, median (IQR), years** | 51.1 (40.1-62.9) | 69.1 (61.3-74.7) | 51.4 (39.7-63.4) | 70.7 (62.8-76.3) |
| **Sex, N (%)** | | | | |
| Male | 1,749,442 (49.8) | 32,789 (46.5) | 1,799,730 (49.8) | 36,853 (46.2) |
| Female | 1,760,013 (50.2) | 37,717 (53.5) | 1,815,067 (50.2) | 42,891 (53.8) |
| **Highest education attainment, N (%)** | | | | |
| ≤9 years | 457,112 (13.0) | 20,822 (29.5) | 440,135 (12.2) | 22,583 (28.3) |
| 10-12 years | 1,586,573 (45.2) | 31,963 (45.3) | 1,612,141 (44.6) | 36,600 (45.9) |
| >12 years | 1,442,351 (41.1) | 17,353 (24.6) | 1,532,603 (42.4) | 20,134 (25.2) |
| Unknown | 23,419 (0.7) | 368 (0.5) | 29,918 (0.8) | 427 (0.5) |
| **Income level, N (%)** | | | | |
| Low | 1,146,954 (32.7) | 41,169 (58.4) | 1,177,585 (32.6) | 47,701 (59.8) |
| Middle | 1,172,085 (33.4) | 16,186 (23.0) | 1,205,956 (33.4) | 17,918 (22.5) |
| High | 1,178,140 (33.6) | 12,975 (18.4) | 1,213,790 (33.6) | 13,926 (17.5) |
| Unknown | 12,276 (0.3) | 176 (0.2) | 17,466 (0.5) | 199 (0.2) |
| **History of psychiatric disorders, N (%)** | | | | |
| No | 3,285,410 (93.6) | 66,573 (94.4) | 2,747,030 (92.3) | 244,934 (93.3) |
| Yes | 224,045 (6.4) | 3933 (5.6) | 228,323 (7.7) | 17,506 (6.7) |
| **History of diabetes, N (%)** | | | | |
| No | 3,453,264 (98.4) | 68,481 (97.1) | 2,941,406 (98.9) | 258,673 (98.6) |
| Yes | 56,191 (1.6) | 2025 (2.9) | 33,947 (1.1) | 3767 (1.4) |
| **History of bereavement, N (%)** | | | | |
| No | 3,247,908 (92.5) | 62,966 (89.3) | 2,838,605 (95.4) | 247,813 (94.4) |
| Yes | 261,547 (7.5) | 7540 (10.7) | 136,748 (4.6) | 14,627 (5.6) |
| **COVID-19 status, N (%)** | | | | |
| No | - | - | 2,048,201 (68.8) | 194,472 (74.1) |
| Yes | - | - | 927,152 (31.2) | 67,968 (25.9) |

© 2026 Yang F et al. *JAMA Netw Open.*

**eTable 6. Incidence rate (per 1000 person-years) and hazard ratio of any cardiovascular disease according to bereavement during the pre-COVID and COVID periods, stratified analyses by sex**

| | Pre-COVID period | | COVID period | |
|---|---|---|---|---|
| | **No. of cases/IR** | **Adjusted HR (95%CI)** [a] | **No. of cases/IR** | **Adjusted HR (95%CI)** [a] |
| **Cohort of partner loss (pre-COVID *vs* COVID period: 3,190,747 *vs* 3,262,204)** | | | | |
| Male (pre-COVID *vs* COVID period: 1,568,367 *vs* 1,600,858) | | | | |
| No loss | 104,914/36.5 | Ref | 103,406/35.2 | Ref |
| Partner loss | 1403/139.6 | 1.34 (1.27-1.41) | 1467/144.4 | 1.48 (1.41-1.56) |
| Female (pre-COVID *vs* COVID period: 1,622,380 *vs* 1,661,346) | | | | |
| No loss | 76,615/25.8 | Ref | 73,482/24.2 | Ref |
| Partner loss | 2227/94.6 | 1.30 (1.24-1.36) | 2433/96.2 | 1.48 (1.42-1.54) |
| **Cohort of child loss (pre-COVID *vs* COVID period: 4,440,584 *vs* 4,537,446)** | | | | |
| Male (pre-COVID *vs* COVID period: 2,053,446 *vs* 2,097,021) | | | | |
| No loss | 144,906/38.4 | Ref | 143,924/37.3 | Ref |
| Child loss | 399/101.7 | 1.30 (1.17-1.43) | 357/91.7 | 1.18 (1.06-1.31) |
| Female (pre-COVID *vs* COVID period: 2,387,138 *vs* 2,440,425) | | | | |
| No loss | 151,119/34.4 | Ref | 145,572/32.3 | Ref |
| Child loss | 663/100.3 | 1.22 (1.13-1.31) | 668/101 | 1.34 (1.24-1.45) |
| **Cohort of parent loss (pre-COVID *vs* COVID period: 3,136,663 *vs* 3,237,793)** | | | | |
| Male (pre-COVID *vs* COVID period: 1,592,826 vs 1,643,745) | | | | |
| No loss | 40,775/14.9 | Ref | 41,202/14.6 | Ref |
| Parent loss | 4027/32.0 | 1.32 (1.28-1.37) | 4189/31.4 | 1.35 (1.31-1.40) |
| Female (pre-COVID *vs* COVID period: 1,543,837 *vs* 1,594,048) | | | | |
| No loss | 33,722/12.7 | Ref | 34,407/12.6 | Ref |
| Parent loss | 3141/24.8 | 1.32 (1.28-1.35) | 3233/24.0 | 1.31 (1.27-1.36) |
| **Cohort of sibling loss (pre-COVID *vs* COVID period: 3,579,961 *vs* 3,694,541)** | | | | |
| Male (pre-COVID *vs* COVID period: 1,782,231 *vs* 1,836,583) | | | | |
| No loss | 101,195/31.3 | Ref | 101,906/30.7 | Ref |
| Sibling loss | 2646/82.6 | 1.15 (1.11-1.20) | 3104/86.0 | 1.18 (1.06-1.31) |
| Female (pre-COVID *vs* COVID period: 1,797,730 *vs* 1,857,958) | | | | |
| No loss | 86,233/26.5 | Ref | 86,066/25.6 | Ref |
| Sibling loss | 2506/67.4 | 1.17 (1.13-1.22) | 3069/72.2 | 1.27 (1.22-1.31) |

IR: incidence rate. HR: Hazard ratio; CI: Confidence interval.

[a] Analyses were adjusted for age, highest educational attainment, household disposable income, and history of diabetes and psychiatric disorders.

Statistically significant differences in the associations between the pre-COVID and COVID periods were observed in the following analyses: for partner loss, the p-value for difference was <0.0001 for female group; and for sibling loss, the p-value for difference was 0.002 for female group. No statistically significant differences were found in the remaining comparisons (p-values > 0.05).

© 2026 Yang F et al. *JAMA Netw Open.*

**eTable 7. Incidence rate (per 1000 person-years) and hazard ratio of any cardiovascular disease according to bereavement during the COVID period, stratified analyses by COVID-19 diagnosis**

| | COVID period | |
|---|---|---|
| | **No. of cases/IR** | **Adjusted HR (95%CI)** [a] |
| **Cohort of partner loss (N= 3,262,204)** | | |
| With COVID-19 (N= 918,285) [b] | | |
| No loss | 41,533/24.2 | Ref |
| Partner loss | 878/147.4 | 1.39 (1.30-1.49) |
| Without COVID-19 (N= 2,343,919) | | |
| No loss | 135,715/31.8 | Ref |
| Partner loss | 3022/102.4 | 1.44 (1.39-1.50) |
| **Cohort of child loss (N=4,537,446)** | | |
| With COVID-19 (N= 1,210,724) [b] | | |
| No loss | 66,069/29.2 | Ref |
| Child loss | 233/113.1 | 1.36 (1.19-1.55) |
| Without COVID-19 (N=3,326,722) | | |
| No loss | 223,427/36.6 | Ref |
| Child loss | 792/93.8 | 1.26 (1.17-1.35) |
| **Cohort of parent loss (N=3,237,793)** | | |
| With COVID-19 (N=950,818) [b] | | |
| No loss | 23,775/13.6 | Ref |
| Parent loss | 1921/27.9 | 1.38 (1.32-1.45) |
| Without COVID-19 (N=2,743,723) | | |
| No loss | 51,834/13.6 | Ref |
| Parent loss | 5501/27.6 | 1.32 (1.28-1.36) |
| **Cohort of sibling loss (N=3,694,541)** | | |
| With COVID-19 (N=995,120) [b] | | |
| No loss | 40,680/23.1 | Ref |
| Sibling loss | 1088/98.2 | 1.30 (1.22-1.38) |
| Without COVID-19 (N=2,242,673) | | |
| No loss | 147,292/29.9 | Ref |
| Sibling loss | 5085/75.3 | 1.22 (1.19-1.26) |

IR: incidence rate. HR: Hazard ratio; CI: Confidence interval.

[a] Analyses were adjusted for age, sex, highest educational attainment, household disposable income, and history of diabetes and psychiatric disorders.

[b] Individuals with COVID-19 included those with a positive PCR test for SARS-CoV-2 recorded in SmiNet, as well as those with a healthcare encounter coded with a COVID-19 ICD-10 diagnosis in the Swedish Patient Register or the Swedish Cause of Death Register.

No statistically significant differences were found in the comparisons between with and without COVID-19 (p-values > 0.05).

**eTable 8. Incidence rate (per 1000 person-years) and hazard ratio of any cardiovascular disease according to bereavement during the pre-COVID and COVID periods, stratified analyses by bereavement history**

| | Pre-COVID period | | COVID period | |
|---|---|---|---|---|
| | **No. of cases/IR** | **Adjusted HR (95%CI)** [a] | **No. of cases/IR** | **Adjusted HR (95%CI)** [a] |
| **Cohort of partner loss (pre-COVID *vs* COVID period: 3,190,747 *vs* 3,262,204)** | | | | |
| With bereavement history (pre-COVID *vs* COVID period: 197,963 *vs* 197,777) | | | | |
| No loss | 13,059/36.2 | Ref | 12,825/35.7 | Ref |
| Partner loss | 187/87.9 | 1.31 (1.13-1.51) | 220/94.8 | 1.43 (1.25-1.63) |
| Without bereavement history (pre-COVID *vs* COVID period: 2,992,784 *vs* 3,064,427) | | | | |
| No loss | 168,471/30.7 | Ref | 164,423/29.2 | Ref |
| Partner loss | 3443/109.4 | 1.30 (1.26-1.35) | 3680/111.1 | 1.47 (1.42-1.52) |
| **Cohort of child loss (pre-COVID *vs* COVID period: 4,440,584 *vs* 4,537,446)** | | | | |
| With bereavement history (pre-COVID *vs* COVID period: 275,603 *vs* 278,238) | | | | |
| No loss | 20,447/40.6 | Ref | 20,300/39.9 | Ref |
| Child loss | 67/92.6 | 1.43 (1.13-1.82) | 72/108.9 | 1.60 (1.27-2.02) |
| Without bereavement history (pre-COVID *vs* COVID period: 4,164,981 *vs* 4,259,208) | | | | |
| No loss | 275,578/35.9 | Ref | 269,196/34.3 | Ref |
| Child loss | 995/101.4 | 1.24 (1.17-1.32) | 953/96.8 | 1.26 (1.19-1.35) |
| **Cohort of parent loss (pre-COVID *vs* COVID period: 3,136,663 *vs* 3,237,793)** | | | | |
| With bereavement history (pre-COVID *vs* COVID period: 149,408 *vs* 151,375) | | | | |
| No loss | 4602/18.5 | Ref | 4565/18.2 | Ref |
| Parent loss | 473/33.6 | 1.33 (1.21-1.47) | 496/32.7 | 1.34 (1.22-1.48) |
| Without bereavement history (pre-COVID *vs* COVID period: 2,987,255 *vs* 3,086,418) | | | | |
| No loss | 69,895/13.6 | Ref | 71,044/13.4 | Ref |
| Parent loss | 6695/28.1 | 1.31 (1.26-1.36) | 6926/27.4 | 1.33 (1.30-1.37) |
| **Cohort of sibling loss (pre-COVID *vs* COVID period: 3,579,961 *vs* 3,694,541)** | | | | |
| With bereavement history (pre-COVID *vs* COVID period: 269,087 vs 271,881) | | | | |
| No loss | 17,656/36.8 | Ref | 17,488/36.2 | Ref |
| Sibling loss | 555/74.1 | 1.20 (1.10-1.31) | 648/79.4 | 1.22 (1.13-1.32) |
| Without bereavement history (pre-COVID *vs* COVID period: 3,310,874 *vs* 3,422,660) | | | | |
| No loss | 169,772/28.3 | Ref | 170,484/27.5 | Ref |
| Sibling loss | 4597/74.5 | 1.16 (1.12-1.19) | 5525/78.4 | 1.24 (1.20-1.27) |

IR: incidence rate. HR: Hazard ratio; CI: Confidence interval.

[a] Analyses were adjusted for age, sex, highest educational attainment, household disposable income, and history of diabetes and psychiatric disorders.

No statistically significant differences were found in the comparisons between with and without bereavement history (p-values > 0.05).

© 2026 Yang F et al. *JAMA Netw Open.*

**eTable 9. Sensitivity analyses for the association between bereavement and the risk of any cardiovascular disease**

| | Pre-COVID period | | COVID period | |
|---|---|---|---|---|
| | **No. of cases/IR** | **Adjusted HR (95%CI)** [a] | **No. of cases/IR** | **Adjusted HR (95%CI)** [a] |
| **Cohort of partner loss (pre-COVID *vs* COVID period: 3,190,747 *vs* 3,262,204)** | | | | |
| Redefined follow-up time [b] | | | | |
| No loss | 181,529/31.0 | Ref | 177,248/29.6 | Ref |
| Partner loss | 3630/110.4 | 1.33 (1.28-1.37) | 3900/112.6 | 1.50 (1.45-1.55) |
| Severe CVD cases [c] | | | | |
| No loss | 52,626/8.8 | Ref | 50,626/8.3 | Ref |
| Partner loss | 1249/35.5 | 1.46 (1.38-1.54) | 1330/35.8 | 1.63 (1.54-1.72) |
| **Cohort of child loss (pre-COVID *vs* COVID period: 4,440,584 *vs* 4,537,446)** | | | | |
| Redefined follow-up time [b] | | | | |
| No loss | 296,025/36.2 | Ref | 289,496/34.6 | Ref |
| Child loss | 1062/104.0 | 1.29 (1.21-1.37) | 1025/100.9 | 1.33 (1.25-1.41) |
| Severe CVD cases [c] | | | | |
| No loss | 93,658/11.2 | Ref | 90,265/10.5 | Ref |
| Child loss | 417/38.0 | 1.40 (1.28-1.55) | 385/35.3 | 1.38 (1.25-1.52) |
| **Cohort of parent loss (pre-COVID *vs* COVID period: 3,136,663 *vs* 3,237,793)** | | | | |
| Redefined follow-up time [b] | | | | |
| No loss | 74,497/13.8 | Ref | 75,609/13.6 | Ref |
| Parent loss | 7168/29.1 | 1.36 (1.32-1.39) | 7422/28.4 | 1.37 (1.34-1.41) |
| Severe CVD cases [c] | | | | |
| No loss | 17,796/3.3 | Ref | 17,665/3.1 | Ref |
| Parent loss | 1871/7.3 | 1.36 (1.29-1.42) | 1985/7.3 | 1.42 (1.35-1.49) |
| **Cohort of sibling loss (pre-COVID *vs* COVID period: 3,579,961 *vs* 3,694,541)** | | | | |
| Redefined follow-up time [b] | | | | |
| No loss | 187,428/28.9 | Ref | 187,972/28.1 | Ref |
| Sibling loss | 5152/77.4 | 1.21 (1.17-1.24) | 6173/82.1 | 1.30 (1.26-1.33) |
| Severe CVD cases [c] | | | | |
| No loss | 54,499/8.2 | Ref | 54,611/8.0 | Ref |
| Sibling loss | 1579/22.0 | 1.16 (1.10-1.22) | 1993/24.5 | 1.26 (1.21-1.32) |

IR: incidence rate. HR: Hazard ratio; CI: Confidence interval; CVD: cardiovascular disease

[a] Analyses were adjusted for age, sex, highest educational attainment, household disposable income, and history of diabetes and psychiatric disorders.

[b] For individuals who experienced more than one loss during the follow-up period, follow-up was censored at the time of the second loss.

[c] We identified CVD cases through the primary discharge diagnosis of an inpatient hospital visit or the underlying cause of death.

Statistically significant differences in the associations between the pre-COVID and COVID periods were observed in the following analyses: for partner loss, the p-values for difference were <0.0001 for restricted bereavement exposure and 0.006 for severe CVD cases; and for sibling loss, the p-values for difference were <0.001 for restricted bereavement exposure and 0.02 for severe CVD cases.

© 2026 Yang F et al. *JAMA Netw Open.*

**eTable 10. Incidence rate (per 1000 person-years) and hazard ratio of any cardiovascular disease according to child loss during the pre-COVID and COVID periods, stratified analyses by number of children in the family**

| | **Pre-COVID period** | | **COVID period** | |
|---|---|---|---|---|
| | **No. of cases/IR** | **Adjusted HR (95%CI)** [a] | **No. of cases/IR** | **Adjusted HR (95%CI)** [a] |
| **Having one child (pre-COVID *vs* COVID period: 959,303 *vs* 976,187)** | | | | |
| No loss | 65,025/36.7 | Ref | 62,847/34.8 | Ref |
| Child loss | 131/108.9 | 1.27 (1.07-1.51) | 140/112.8 | 1.32 (1.12-1.56) |
| **Having two children (pre-COVID *vs* COVID period: 2,176,001 *vs* 2,229,211)** | | | | |
| No loss | 136,932/34.1 | Ref | 134,066/32.5 | Ref |
| Child loss | 431/102.6 | 1.28 (1.16-1.40) | 404/100.5 | 1.34 (1.22-1.48) |
| **Having more than two children (pre-COVID *vs* COVID period: 1,305,280 *vs* 1,332,048)** | | | | |
| No loss | 94,068/39.4 | Ref | 92,583/38.0 | Ref |
| Child loss | 500/97.4 | 1.22 (1.12-1.33) | 481/91.6 | 1.22 (1.11-1.33) |

IR: incidence rate. HR: Hazard ratio; CI: Confidence interval; CVD: cardiovascular disease

[a] Analyses were adjusted for age, sex, highest educational attainment, household disposable income, and history of diabetes and psychiatric disorders.

© 2026 Yang F et al. *JAMA Netw Open.*

**eTable 11. Incidence rate (per 1000 person-years) and hazard ratio of any cardiovascular disease according to sibling loss during the pre-COVID and COVID periods, stratified analyses by number of siblings in the family**

| | Pre-COVID period | | COVID period | |
|---|---|---|---|---|
| | **No. of cases/IR** | **Adjusted HR (95%CI)** [a] | **No. of cases/IR** | **Adjusted HR (95%CI)** [a] |
| **Having one sibling (pre-COVID *vs* COVID period: 1,802,915 *vs* 1,877,957)** | | | | |
| No loss | 92,826/28.0 | Ref | 95,065/27.5 | Ref |
| Sibling loss | 1337/75.9 | 1.12 (1.07-1.19) | 1673/82.3 | 1.21 (1.15-1.27) |
| **Having two siblings (pre-COVID *vs* COVID period: 1,100,601 *vs* 1,139,031)** | | | | |
| No loss | 55,279/27.7 | Ref | 55,268/26.8 | Ref |
| Sibling loss | 1526/75.8 | 1.23 (1.16-1.29) | 1840/79.3 | 1.31 (1.25-1.38) |
| **Having more than two siblings (pre-COVID *vs* COVID period: 676,445 *vs* 677,553)** | | | | |
| No loss | 39,323/33.8 | Ref | 37,639/32.5 | Ref |
| Sibling loss | 2289/72.8 | 1.18 (1.13-1.23) | 2660/75.8 | 1.25 (1.20-1.30) |

IR: incidence rate. HR: Hazard ratio; CI: Confidence interval; CVD: cardiovascular disease

[a] Analyses were adjusted for age, sex, highest educational attainment, household disposable income, and history of diabetes and psychiatric disorders.

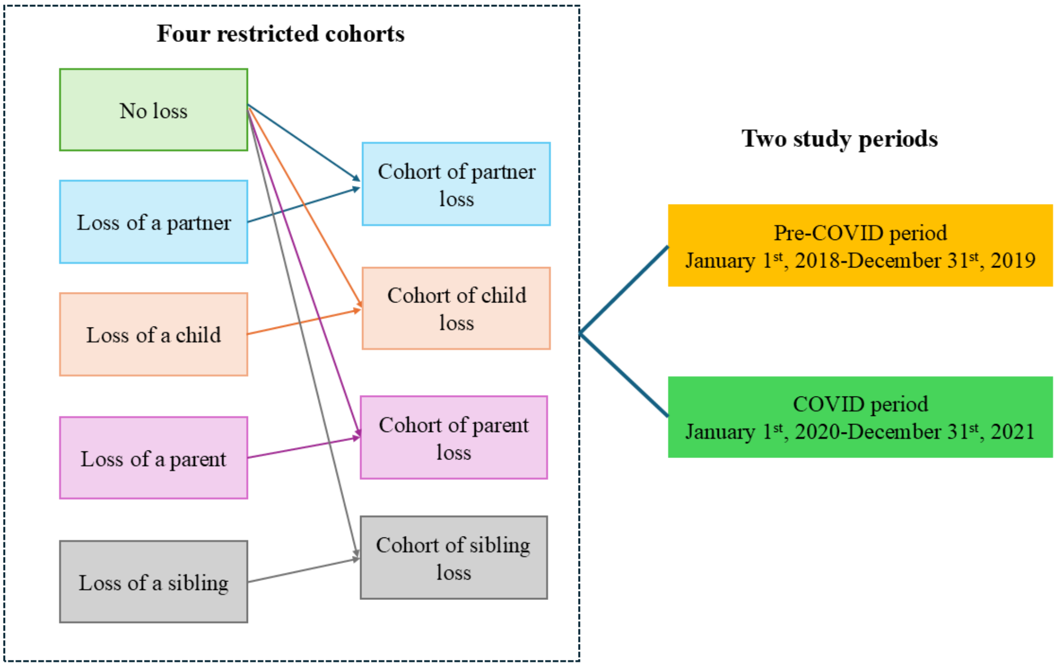


**eFigure 1. Study design**

© 2026 Yang F et al. *JAMA Netw Open.*

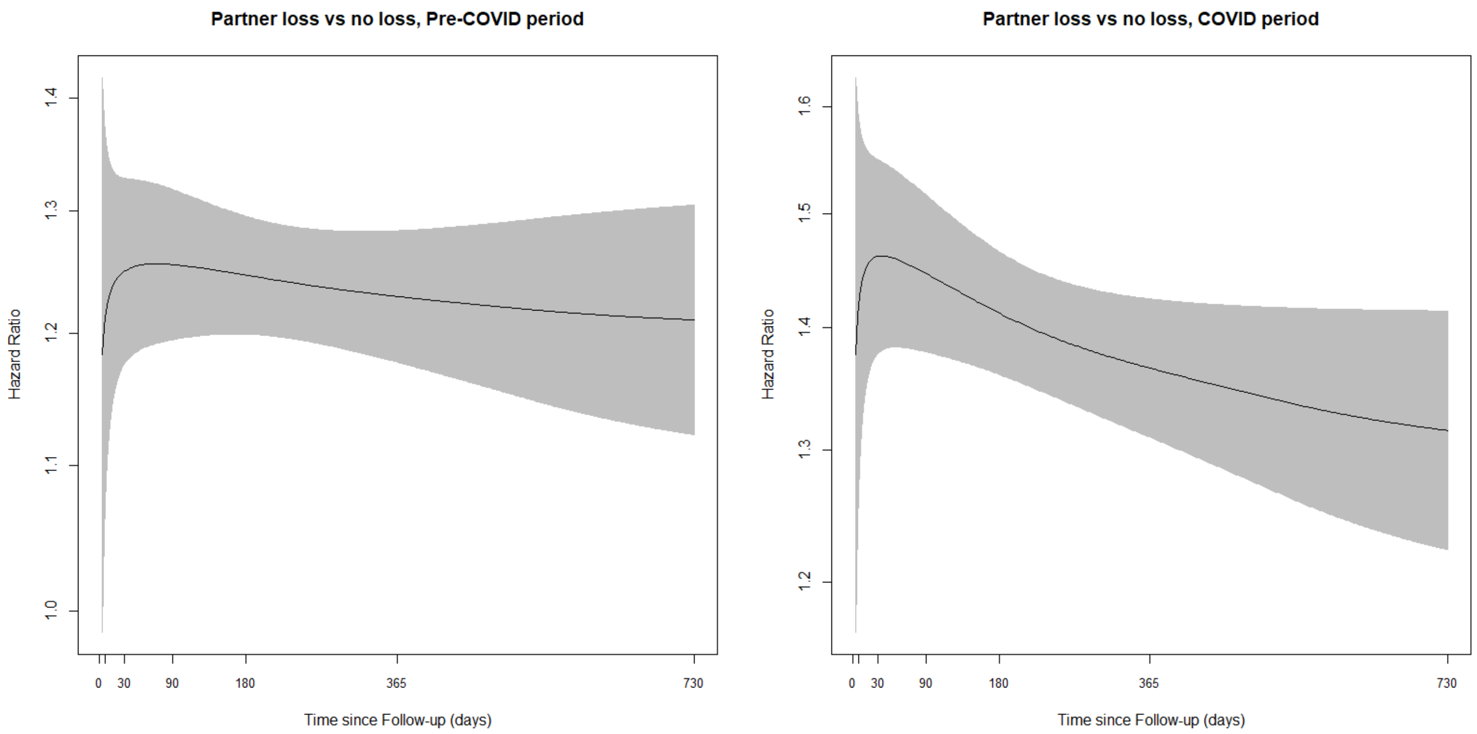


**eFigure 2. Hazard ratios of any cardiovascular disease in relation to partner loss, analyses using flexible parametric survival models.** The flexible parametric survival model was implemented using the R package rstpm2. The baseline hazard was modeled with a restricted cubic spline with 5 degrees of freedom (4 interior knots and 2 boundary knots), with knots placed at the quintiles of the event-time distribution. Time-varying effects were modeled using a restricted cubic spline with 3 degrees of freedom. The model was adjusted for age, sex, highest educational attainment, household disposable income, and history of diabetes and psychiatric disorders.

© 2026 Yang F et al. *JAMA Netw Open.*

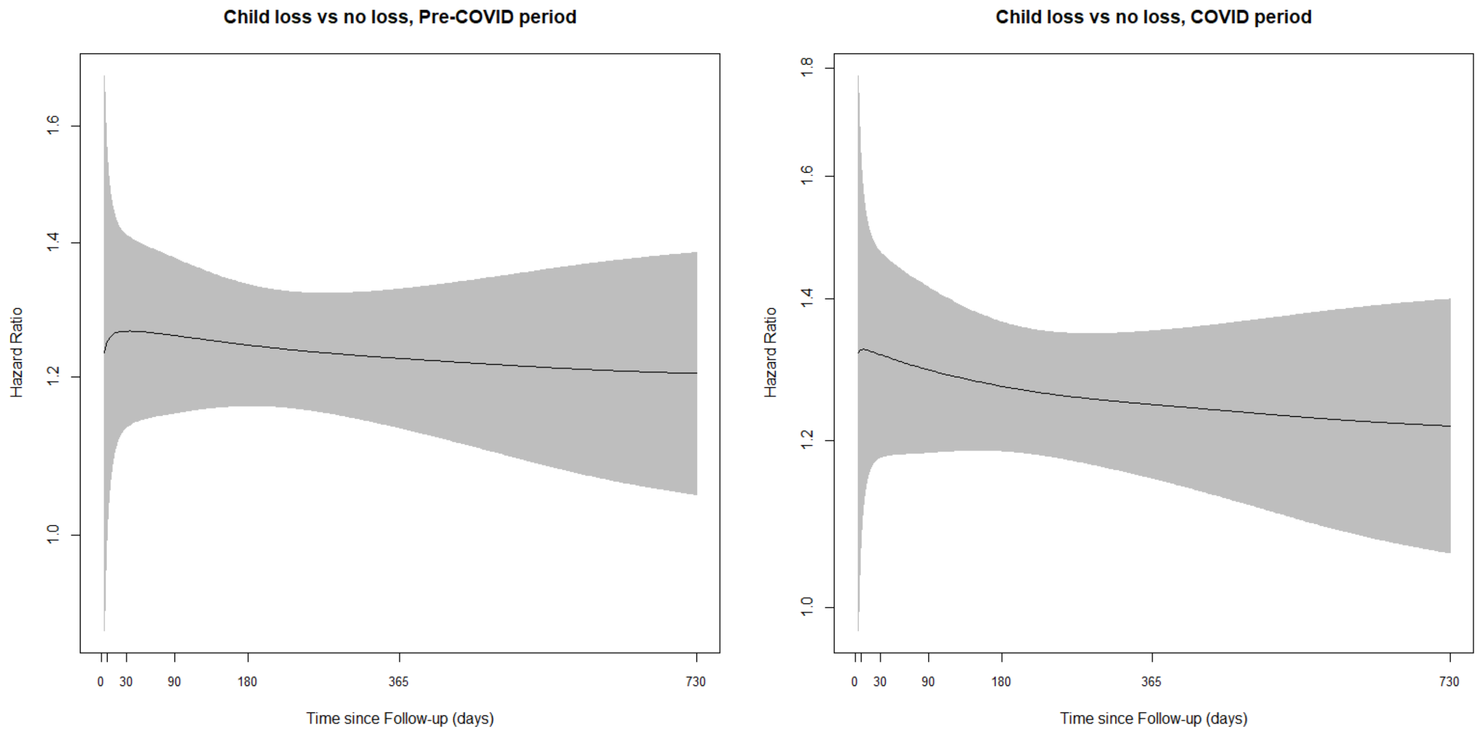


**eFigure 3. Hazard ratios of any cardiovascular disease in relation to child loss, analyses using flexible parametric survival models.** The flexible parametric survival model was implemented using the R package rstpm2. The baseline hazard was modeled with a restricted cubic spline with 5 degrees of freedom (4 interior knots and 2 boundary knots), with knots placed at the quintiles of the event-time distribution. Time-varying effects were modeled using a restricted cubic spline with 3 degrees of freedom. The model was adjusted for age, sex, highest educational attainment, household disposable income, and history of diabetes and psychiatric disorders.

© 2026 Yang F et al. *JAMA Netw Open.*

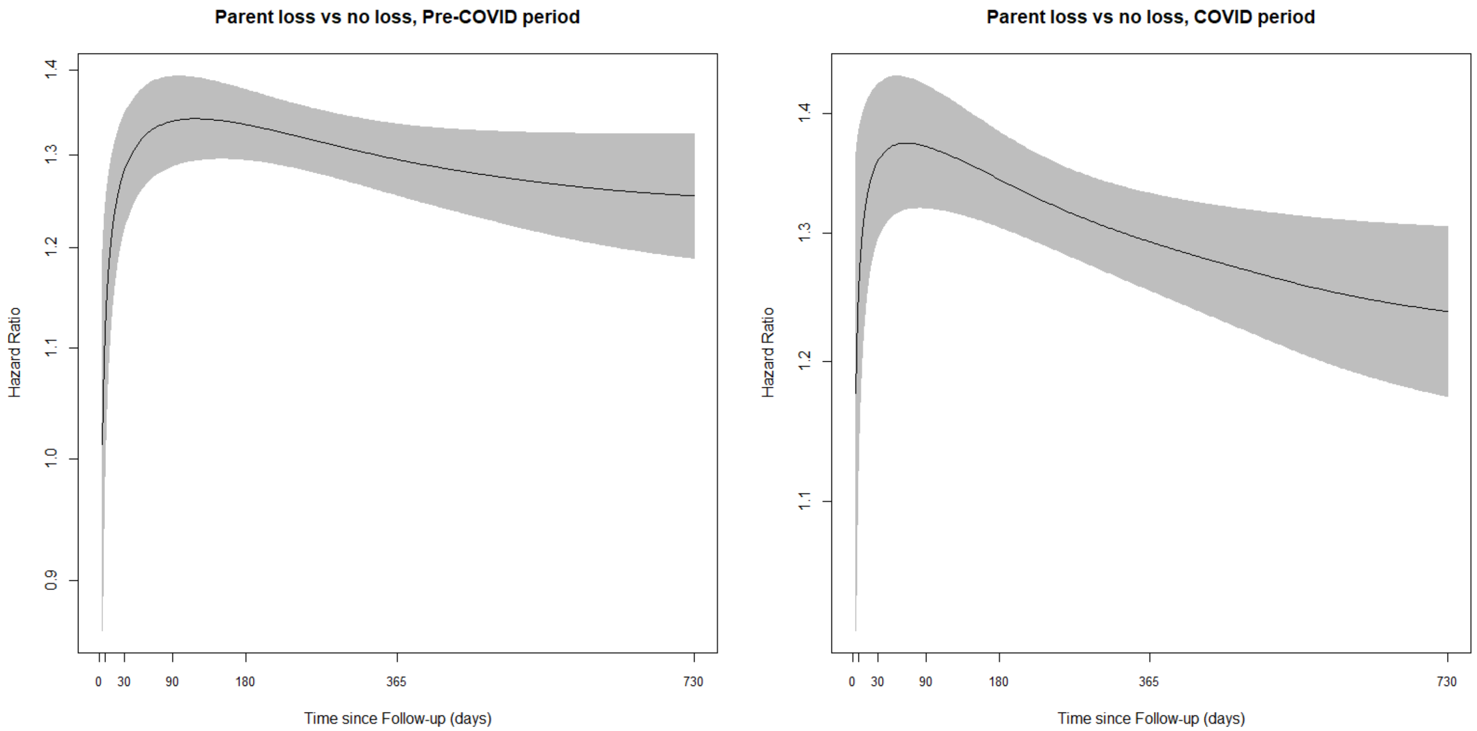


**eFigure 4. Hazard ratios of any cardiovascular disease in relation to parent loss, analyses using flexible parametric survival models.** The flexible parametric survival model was implemented using the R package rstpm2. The baseline hazard was modeled with a restricted cubic spline with 5 degrees of freedom (4 interior knots and 2 boundary knots), with knots placed at the quintiles of the event-time distribution. Time-varying effects were modeled using a restricted cubic spline with 3 degrees of freedom. The model was adjusted for age, sex, highest educational attainment, household disposable income, and history of diabetes and psychiatric disorders.

© 2026 Yang F et al. *JAMA Netw Open.*

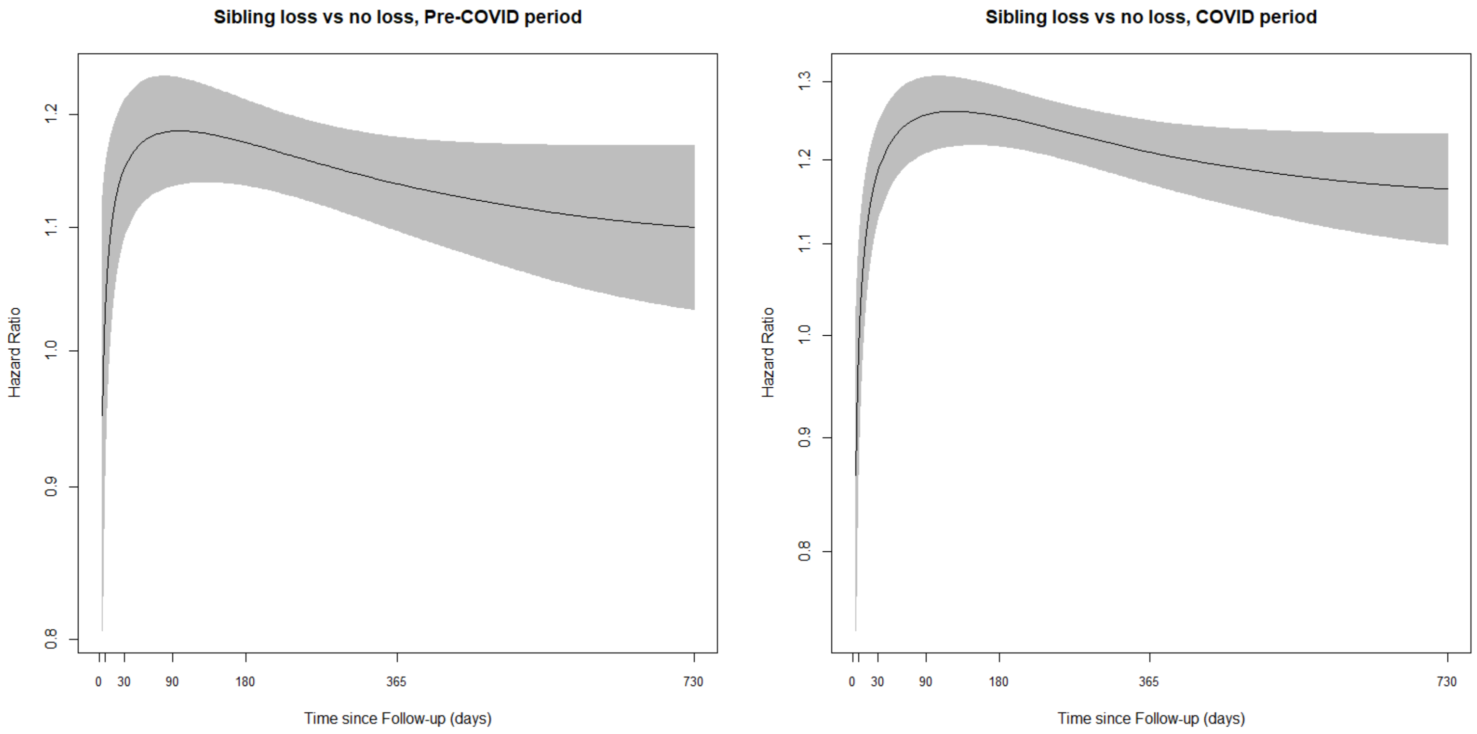


**eFigure 5. Hazard ratios of any cardiovascular disease in relation to sibling loss, analyses using flexible parametric survival models.** The flexible parametric survival model was implemented using the R package rstpm2. The baseline hazard was modeled with a restricted cubic spline with 5 degrees of freedom (4 interior knots and 2 boundary knots), with knots placed at the quintiles of the event-time distribution. Time-varying effects were modeled using a restricted cubic spline with 3 degrees of freedom. The model was adjusted for age, sex, highest educational attainment, household disposable income, and history of diabetes and psychiatric disorders.

© 2026 Yang F et al. *JAMA Netw Open.*

**eAppendix 1. Description of the source, the measurement and the categorization of covariates**

Covariates included age at the start of each study period, sex (male or female), highest educational attainment (≤9 years, 10-12 years, or >12 years), disposable household income (categorized according to the tertile distribution of each restricted cohort: low, middle, or high), diagnosis of COVID-19 during the follow-up period (only available for the COVID period), previous bereavement experience (retrieved from the two years preceding the study period), and diagnoses of diabetes and psychiatric within the three years preceding the study period (given data availability). Data on age and sex were obtained from the Swedish Total Population Register, while information on education and disposable income was extracted from the Longitudinal Integration Database for Health Insurance and Labour Market Studies (LISA).[1] COVID-19 diagnosis was identified through SmiNet[2]—the national register of notifiable communicable diseases managed by the Public Health Agency of Sweden, which records individuals with positive SARS-CoV-2 PCR test results—or through the Swedish Patient Register and the Swedish Causes of Death Register, using relevant ICD codes (eTable 1 in the Supplement). Data on history of diabetes and psychiatric disorders were extracted from the Swedish Patient Register, including both primary and secondary diagnoses from inpatient and outpatient visits (eTable 1 in the Supplement).

**eReferences**


1. Ludvigsson, J.F., Svedberg, P., Olén, O., Bruze, G. & Neovius, M. The longitudinal integrated database for health insurance and labour market studies (LISA) and its use in medical research. *Eur J Epidemiol* **34**, 423-437 (2019).
2. Rolfhamre, P., Jansson, A., Arneborn, M. & Ekdahl, K. SmiNet-2: Description of an internet-based surveillance system for communicable diseases in Sweden. *Euro Surveill* **11**, 103-107 (2006).


© 2026 Yang F et al. *JAMA Netw Open.*
