# Supplementary material for: Bereavement and Risk of Cardiovascular Disease Before and During the COVID-19 Pandemic
Source: JAMA Netw Open. 2026 Apr 24;9(4):e269102. doi: 10.1001/jamanetworkopen.2026.9102 (PMC13109797; doi:10.1001/jamanetworkopen.2026.9102)
Supplement: Supplement 2. — Data Sharing Statement [file jamanetwopen-e269102-s002.pdf]

Data Sharing Statement

Yang. Bereavement and Risk of Cardiovascular Disease Before and During the COVID-19 Pandemic. *JAMA Netw Open*. Published April 24, 2026.
doi:10.1001/jamanetworkopen.2026.9102

Data
Data available: No

Additional Information
Explanation for why data not available: We are not allowed to share data from the Swedish registers due to strict regulation and ethical considerations.
